# Vaccine Therapy and Sanatoria

**Published:** 1912-05-25

**Authors:** 


### VACCINE THERAPY AND SANATORIA.

### The Inoculation Department of St. Mary's Hospital.

From the inoculation department of St. Mary's Hospital there has been recently issued an interesting report, couched in more or less popular language, explaining the scope of the work done in this department by Sir Almroth Wright and his assistants.

The department of therapeutic immunisation was reconstructed in 1909 under a committee of which the Right Hon. A. J. Balfour was the chairman, and the work which was begun in 1903 in one small room is now carried on in the Clarence Wing of St. Mary's Hospital with the enormous advantage of having a certain number of beds available for the study and treatment of patients undergoing vaccine therapy. In the course of the report a useful and lucid exposition of the *rationale* and some hints of the technique of vaccine treatment is given; this should enable the intelligent layman to appreciate more fully not only the expectations and limitations of the work, but also the difficulties with which these scientific investigators have to contend. After enlarging upon the simile which compares this treatment to the weeding of a garden, the special researches which are still being carried on are mentioned in some detail with illustrative cases. Considerable space is devoted to the subject of tubercle in its many forms, and this portion of the report will, at the present time, be studied with interest. The general impression given is that satisfactory progress has been made and that as more work is done it will be found that more and more cases are amenable to treatment by bacillary vaccines.

But it must be carefully borne in mind that such treatment is not in any sense intended as a substitute for sanatorium treatment, but rather as a supplementary method for providing more accurate dosage than by the other method of auto-inoculation produced by graduated exercises. Hygienic surroundings and constant medical supervision are as important in the vaccine or tuberculin treatment as in the cases of patients undergoing the *régime* of a sanatorium, though a certain number of cases treated as out-patients and living under unhygienic conditions have been noted by various observers to have responded well and quickly to the inoculations of a tubercle vaccine. The report deals also with the problems of chronic joint diseases, so called indigestion, oral sepsis, and other common ailments which disable so many men and women in what should be their prime of life and which are unfortunately so common amongst those who can least afford to be in ill-health. In these directions progress must necessarily be slow owing to the difficulty of tracking the causal organism, but results have already been obtained sufficiently good to justify the hope that the "pleasing potentialities" referred to will mature into therapeutic triumphs by-and-by.

